# Autoimmune/Inflammatory Syndrome Induced by Adjuvants and Thyroid Autoimmunity

**DOI:** 10.3389/fendo.2016.00150

**Published:** 2017-01-24

**Authors:** Abdulla Watad, Paula David, Stav Brown, Yehuda Shoenfeld

**Affiliations:** ^1^Department of Medicine “B”, Sheba Medical Center, Tel-Aviv, Israel; ^2^Zabludowicz Center for Autoimmune Diseases, Sheba Medical Center, Tel-Aviv, Israel; ^3^Sackler Faculty of Medicine, Tel-Aviv University, Tel-Aviv, Israel; ^4^Faculdade de Ciências Médicas, Universidade do Estado do Rio de Janeiro, Rio de Janeiro, Brazil; ^5^Laura Schwarz-Kipp Chair for Research of Autoimmune Diseases, Tel-Aviv University, Tel-Aviv, Israel

**Keywords:** autoimmune/inflammatory syndrome induced by adjuvants, thyroid, endocrinopathy, adjuvants, vaccines, silicone, Hashimoto’s thyroiditis, Graves disease

## Abstract

The autoimmune/inflammatory syndrome induced by adjuvants (ASIA), presented by Shoenfeld and Agmon-Levin in 2011, is an entity that incorporates diverse autoimmune conditions induced by the exposure to various adjuvants. Adjuvants are agents that entail the capability to induce immune reactions. Adjuvants are found in many vaccines and used mainly to increase the response to vaccination in the general population. Silicone has also been reported to be able to induce diverse immune reactions. Clinical cases and series of heterogeneous autoimmune conditions including systemic sclerosis, systemic lupus erythematosus, and rheumatoid arthritis have been reported to be induced by several adjuvants. However, only a small number of cases of autoimmune thyroid disorder have been included under the umbrella of ASIA syndrome. Indeed, clinical cases of Hashimoto’s thyroiditis and/or subacute thyroiditis were observed after the exposure to vaccines as well as silicone implantation. In our review, we aimed to summarize the current knowledge on ASIA syndrome presented as endocrinopathies, focusing on autoimmune thyroid disorders associated with the various adjuvants.

## Introduction

Adjuvants are substances that are able to trigger autoimmunity *via* a variety of mechanisms, such as alteration of the host’s immune system, polyclonal activation of B cells, effects on cellular immunity, immunoregulatory cells, viral-induced antibodies, and acceleration of molecular mimicry (1). Exposure to adjuvants can occur in a variety of methods due to their wide range of uses in vaccines, mineral oils, silicone implants, and many other products and devices. The association between adjuvant exposure and autoimmunity manifests itself in five autoimmune conditions sharing similar autoimmunity manifestations (2, 3), such as the postvaccination phenomena, the macrophagic myofasciitis syndrome (MMF), the Gulf war syndrome (GWS), siliconosis, and the sick building syndrome (SBS) (4, 5). The autoimmune/inflammatory syndrome induced by adjuvants (ASIA), presented by Shoenfeld and Agmon-Levin (6) in 2011, is a single entity that incorporates all five conditions. Extensive research has identified the genetic background, contributing to the development of ASIA syndrome in predisposed individuals following adjuvant exposure. A large number of autoimmune diseases share several alleles of the HLA class II such as DRB1 locus. The development of specific autoantibodies is determined by DRB1 alleles leading to an abnormal response and development of full-blown autoimmune diseases (7, 8).

When used in vaccines, adjuvants are purposely used as immunogenicity enhancing agents that are essential for directing the adaptive immunoresponse (9). However, they might also trigger undesired autoimmune reactions that question the use of adjuvants and their safety in the context of DRB1*01 genetic background (10).

A systematic review by Jara et al. (4) reported that 4479 ASIA cases have been identified since its presentation in 2011. Among them, 305 were considered severe, with the majority of these cases being developed following vaccines mainly directed to HPV, HBV, and seasonal influenza. Despite vaccines’ proven record of safety and efficiency, aluminum hydroxide was used in these vaccines along with the viral antigens as an adjuvant. Due to aluminum’s capability to enhance the immunoresponse, it enables the usage of smaller amount of antigens. However, enhanced immunogenicity might lead to enhanced reactogenicity in a process not always benign involving pathological stimulation (11).

The other adjuvants containing products yielding severe clinical manifestations are silicone implants and mineral oil fillers (4, 12).

Silicone has been considered as an inert material, which is unable to induce immune reactions in the human body. Therefore, it has been used in many medical devices for the last 60 years, including both silicone and saline breast implants. However, a possible association between silicone exposure and autoimmune diseases has been reported in many studies demonstrating the development of autoimmune diseases and autoantibodies in patients following exposure to silicone implants (13, 14). Improved clinical manifestations after the extraction of implants (15) support the relationship between silicone and autoimmunity.

Mineral oil injections, which are prevalent in Mexico and Latin America for cosmetic uses, have been identified as a leading cause of ASIA syndrome as well *via* the proposed mechanism of chronic inflammation induction leading to granuloma formation and thickening of the dermis (4, 10).

The risk for autoimmune diseases, determined by the patient’s genetic background, is increased in patients with autoimmune diseases history such as type 1 diabetes mellitus (T1DM). Thyroid antibodies can be identified in approximately 20–25% of patients with type 1 diabetes, and up to 50% of them progress to clinical autoimmune thyroid disease (AITD) (16). Thyroid autoimmune diseases have been described in many case reports and case series, presenting thyroid autoimmune manifestations along with other autoimmune conditions.

In genetically predisposed individuals, under particular conditions, molecular mimicry between microbial and human antigens has been shown to be able to turn a defensive immunoresponse into autoimmune response. This mechanism has yet to be explored in the field of thyroid autoimmune diseases (17). In our review, we aimed to summarize the current knowledge about ASIA syndrome and the relationship between adjuvants and autoimmune diseases, focusing on its association with autoimmune endocrinopathies and thyroid autoimmunity.

## Endocrinopathy and ASIA Syndrome

Pathological processes of the endocrine glands result in abnormal levels of circulating hormones, which lead to endocrinopathies. Some endocrine disorders are immune mediated, such as Hashimoto’s thyroiditis (HT), Graves’ disease, and T1DM (18–20). Thus, it is possible that endocrine autoimmune diseases can be triggered by adjuvants, configuring cases of ASIA syndrome. Case reports, cohort and case–control studies on ASIA syndrome, and the majority of the endocrinopathies are still scarce. Lately, primary ovarian failure (POF) has been linked to ASIA, especially after vaccination (21–25).

Primary ovarian failure or premature ovarian insufficiency is defined as a combination of amenorrhea, for a minimum of 4 months, decline in sex steroids, and follicle-stimulating hormone (FSH) above 40 IU/l at two measurements with an interval of at least 1 month in women younger than 40 years (26). POF is a disorder with multiple etiologic mechanisms. The presence of lymphocytic invasion in the oophorus and the identification of autoantibodies against ovarium antigens on the theca, granulose, corpus luteum, and zona pellucida (27–29) support the idea that part of its etiology, estimated in 20–30% (30), is immune mediated. Furthermore, POF is commonly associated with other autoimmune diseases, including Addison’s disease, thyroiditis, autoimmune polyglandular syndrome, systemic lupus erythematosus (SLE), hemolytic anemia, idiopathic thrombocytopenic purpura (ITP), and Sjogren’s syndrome (31). The pathogenesis of POF also involves genetic mutations, metabolic disorders, and environmental factors, such as virus infection, chemo and radiotherapy, and surgeries (30).

HPV vaccine has been reported as an important issue in ASIA syndrome, already being related, for instance, to Guillain-Barré syndrome and other neuropathies, such as SLE, vasculitis, ITP, and autoimmune hepatitis (32–36). Developing autoimmune diseases as an adverse effect of the vaccine can be both due to its HPV virus-like particles, which have potent immuno-stimulatory properties (and can induce autoimmunity by molecular mimicry, epitope spreading, bystander activation, and polyclonal activation) (37), and due to the presence of aluminum as an adjuvant in the vaccine (38). Adjuvants are capable of increasing, intensifying, and prolonging antigen-specific immunoresponse of the vaccines without holding its own specific antigenic effect (38). Autoimmune well-defined diseases, as well as the non-specific immune disorders, following vaccination can present as a subacute vaccination side effect or appear months or years after the boosters (39–43). Genetically predisposed patients are more likely to exhibit late manifestations and are in a higher risk of developing ASIA syndrome (36, 44).

Colafrancesco et al. (21) recently reported three cases of POF following immunization with HPV vaccine. The three patients fulfilled the criteria for ASIA syndrome suggested by Shoenfeld and Agmon-Levin (6). They described three young women, previously healthy and with normal sexual development, who received three administrations of the quadrivalent HPV vaccine. The patients experienced general symptoms, including nausea, stomachaches, heavy and burning sensations in the injected arm, headaches, insomnia, arthralgia, depression, anxiety, and difficulty in concentrating, and then presented amenorrhea within approximately 10 months, 2 years, and 10 years after the first dose. Two of them were positive for previously negative antibodies (anti-TPO and antiovarian). Hormonal screening was performed, showing increased FSH and luteinizing hormone (LH) plus extremely low levels of estradiol. Pregnancy was excluded, as well as no abnormalities were revealed in the transvaginal and pelvic ultrasound. After a karyotype evaluation and search for Fragile X syndrome with no aberrations, they were diagnosed with POF. Moreover, two of the three patients were siblings leading to the hypothesis that may exist as a rare risk factor for this adverse effect.

Little and Ward (22) also reported a case of POF succeeding HPV vaccination, in a 16-year-old patient, who presented irregular menses after taking the quadrivalent vaccine, followed by oligomenorrhea and amenorrhea. Her hormone profile also showed high levels of FSH and LH and low levels of estradiol and anti-mullerian hormone (AMH), and after excluding pregnancy and genetic, endocrinal, and other causes, she was diagnosed with POF.

Problems of quadrivalent HPV vaccine introduction in the market were wisely pointed by Little and Ward (25). They reported three other cases of young women who develop POF after having quadrivalent HPV vaccine and questioned some issues about its safety. First, despite the fact that the vaccine protocol suggests three doses, in the preclinical studies for toxicity, only two boosters were given to the rats. Still, the animals’ reproductive system was not analyzed in a long-term period. Moreover, the phase II and III clinical studies on safety of the vaccine regarding the fertility were not complete: half of the subjects studied were lost to follow-up at 1 year; some of the subjects were on hormone contraception methods, which could mask the ovarian insufficiency; they have not considered medical conditions that flourished more than 7 months after the vaccination as associated with the vaccine; and adverse effects were only reported 2 weeks after the boosters. Furthermore, the placebo used as control in the phase III safety studies of the quadrivalent HPV vaccine was aluminum, also present in the vaccine solution, which was already shown to play as an adjuvant in ASIA syndrome.

Thus, HPV vaccine is likely to be an important trigger in ASIA syndrome, including immuno-mediated endocrine disorders, such as POF. Due to long periods of intervals between the vaccine injections and the development of the ovarian insufficiency, it is questionable if there is indeed a causal relationship between them. However, as previously mentioned, the safety preclinical and clinical studies of HPV vaccine are lacking some information regarding fertility safety, and the side effects were shown to be able to appear even after months or years.

Other vaccines and adjuvants may also trigger POF, as well as other immuno-mediated endocrinopathies, like for instance, type 1-diabetes may be induced by the same adjuvants. Indeed, in a cohort study with 211 young female patients with autoimmune diseases and 857 matched controls, they showed that patients exposed to quadrivalent HPV vaccine were in a higher risk of developing type 1-diabetes mellitus (OR = 1.2) (45). Additionally, it was shown in a prospective cohort study (46) that some vaccines are related to increased levels of diabetes autoantibodies, such as antibody against glutamic acid decarboxylase (GADA) and tyrosine phosphatase (IA-2A). These autoantibodies, which are considered reliable markers for the disease process (47, 48), were more frequently found in the subjects who received hemophilus influenza B (HIB) vaccination (OR = 5.9 and 3.4 in IA-2A and GADA, respectively). Especially, the IA-2A serum concentrations were significantly higher in patients exposed to HIB. Also, BCG was correlated to an enhanced prevalence of IA-2A (*p* < 0.01). The previously mentioned studies suggest that ASIA syndrome, particularly post vaccination, and endocrinopathies might be linked.

## Autoimmune Thyroid Disease and ASIA Syndrome

During the last years, abundant case reports and series were published supporting that various autoimmune disorders may be induced by adjuvants and be enclosed under ASIA syndrome (4, 12). Despite the fact of being the most common autoimmune disorder, unexpectedly, we have revealed very few articles and case reports in the literature describing the induction of AITD by various adjuvants. In this section, we report that the relevant case descriptions of AITD were reported to be correlated to immunization and silicone implants.

Hernàn Martinez et al. (49) described a case of a 55-year-old man with a family history of autoimmune diseases and medical history of diabetes and psoriasis, who developed subacute thyroiditis shortly after the administration of an influenza vaccine. Subacute thyroiditis is a very rare disease, and the authors of the mentioned case concluded that the induction of the disease was a result of an interaction between the genetic predisposition and vaccination. Another similar case of subacute thyroiditis was reported in a 25-year-old female (50). The patient was admitted due to fever, swelling, and tender mass in the neck. Two days before her presentation, she received influenza vaccine (Vaxigrip). Biopsy of the thyroid has revealed multinuclear giant cell granulomas.

A previously healthy 36-year-old female presented with clinical symptoms of thyrotoxicosis including tachycardia, anxiety, and tenderness in her neck (51). One month before her presentation, she received H1N1 vaccine. Thyroid function tests confirmed remarkable thyrotoxicosis. Thyroid scintigraphy was performed and showed significant diffuse reduction in the technetium uptake. Therefore, a diagnosis of subacute thyroiditis was made. Moving to another type of adjuvant, cases of granulomatous inflammation of the thyroid have been reported with silicone breast implants (52). Vayssairat et al. (53) described two cases of HT after receiving a silicone gel-filled breast implants. Both cases were induced after a long period of incubation, the first case is a 45-year-old women who had bilateral silicone implant of the breast in 1976 and developed HT in 1991. In addition, the patient complained of other non-specific symptoms including fatigue, morning stiffness, and sicca syndrome. Thyroid ultrasonography showed an enlarged thyroid gland with a diffusely hypoechogenic pattern. The implants were painful and removed, showing extremely dense connective tissue with fibrosis. The second case of HT presented with hyperthyroidism clinical manifestation, 10 years after the silicon implantation, reporting positive anti-TPO. The implants were again painful, and the patient developed positive antinuclear antibodies (ANA). An animal experiment aimed to evaluate the immunological adjuvancy potential of silicone gel taken from breast implants (54). The study has found that silicone gel is able to stimulate the production of autoantibodies to rat thyroglobulin and bovine collagen II. However, this immune reaction was not associated with any histological evidence of thyroiditis or arthritis.

A cohort study was performed to assess the risk of new onset autoimmune disease in young women exposed to human papillomavirus-16/18 AS04-adjuvanted vaccine in the United Kingdom (55). The study reported an incidence rate ratio (95% CI) of 3.75 (1.25–11.31) for autoimmune thyroiditis among females.

An animal study has reported that immunization of BALB/c mice with the extracellular domain of the human TSH receptor led to the production of TSH binding-inhibiting and thyroid-blocking antibodies accompanied by lymphocytic infiltration of the thyroid (56).

In summary, ASIA syndrome is being more recognized by physicians, and therefore, more studies and cases have reported the correlation of the exposure to various adjuvants with diverse autoimmune diseases. Still, very few clinical reports and animal models studies were published to show the relationship between endocrinopathies in general and AITD in particular with adjuvants. However, the clinical cases of HT and/or subacute thyroiditis were observed after the exposure to vaccines as well as silicone implantation. Therefore, we believe that the minority of cases is not owing to rarity of association between adjuvants and AITD rather than the lack of awareness among physicians of such association. Consequently, physicians must be mindful that thyroiditis and other thyroid disorders can be induced by diverse adjuvants and therefore to reconsider non-essential vaccination in genetically predisposed individuals for autoimmune diseases.

## Author Contributions

AW, PD, SB, and YS designed the study and reviewed the literature on ASIA syndrome and thyroid autoimmunity. AW, PD, and SB wrote the manuscript. AW and YS edited the manuscript. All the authors have revised the paper and approved the final edition.

## Conflict of Interest Statement

The authors declare that the research was conducted in the absence of any commercial or financial relationships that could be construed as a potential conflict of interest. The reviewers IR and SF and handling Editor declared their shared affiliation, and the handling Editor states that the process nevertheless met the standards of a fair and objective review.
